# Catalysis of Protein Folding by Chaperones Accelerates Evolutionary Dynamics in Adapting Cell Populations

**DOI:** 10.1371/journal.pcbi.1003269

**Published:** 2013-11-07

**Authors:** Murat Çetinbaş, Eugene I. Shakhnovich

**Affiliations:** Department of Chemistry and Chemical Biology, Harvard University, Cambridge, Massachusetts, United States of America; Stanford University, United States of America

## Abstract

Although molecular chaperones are essential components of protein homeostatic machinery, their mechanism of action and impact on adaptation and evolutionary dynamics remain controversial. Here we developed a physics-based ab initio multi-scale model of a living cell for population dynamics simulations to elucidate the effect of chaperones on adaptive evolution. The 6-loci genomes of model cells encode model proteins, whose folding and interactions in cellular milieu can be evaluated exactly from their genome sequences. A genotype-phenotype relationship that is based on a simple yet non-trivially postulated protein-protein interaction (PPI) network determines the cell division rate. Model proteins can exist in native and molten globule states and participate in functional and all possible promiscuous non-functional PPIs. We find that an active chaperone mechanism, whereby chaperones directly catalyze protein folding, has a significant impact on the cellular fitness and the rate of evolutionary dynamics, while passive chaperones, which just maintain misfolded proteins in soluble complexes have a negligible effect on the fitness. We find that by partially releasing the constraint on protein stability, active chaperones promote a deeper exploration of sequence space to strengthen functional PPIs, and diminish the non-functional PPIs. A key experimentally testable prediction emerging from our analysis is that down-regulation of chaperones that catalyze protein folding significantly slows down the adaptation dynamics.

## Author Summary

Molecular chaperones or heat-shock proteins are essential components of protein homeostatic machinery in all three domains of life, whose role is not only to prevent protein aggregation but also catalyze the protein folding process by decreasing the energetic barrier for folding. Importantly, chaperones have often been implicated as phenotypic capacitors since they buffer the deleterious effects of mutations, promote genetic diversity, and thus speed up adaptive evolution. Here we explore computationally the consequences of chaperone activity in cytoplasm via long-time evolutionary dynamics simulations. We use a 6-loci multi scale model of cell populations, where the fitness of each cell is determined from its genome, based on statistical mechanical principles of protein folding and protein-protein interactions. We find that by catalyzing protein folding chaperones buffer the deleterious effect of mutations on folding stability and thus open up a sequence space for efficient and simultaneous optimization of multiple molecular traits determining the cellular fitness. As a result, chaperones dramatically accelerate adaptation dynamics.

## Introduction

Evolutionary selection of protein sequences is a complex task whereby several traits such as translation efficiency, structural integrity (i.e. folding stability and kinetics), molecular function, as well as interactions with other proteins in the cellular milieu should be simultaneously optimized. Imposing simultaneous and often contradictive (pleiotropic) constraints on protein sequence evolution severely limits the repertoire of possible solutions in sequence space and thus slows down the evolutionary dynamics. It is widely accepted that strong selective pressure against protein misfolding plays a key role in determining the rate of protein evolution and sustainable mutational loads [1]–[5]. However, other constraints such as the need to avoid protein sequestration to non-functional protein-protein interactions (NF-PPIs) in the cytoplasm are also emerging as important determinants of the rates and outcomes of evolutionary dynamics of proteins [6]–[10].

From *de novo* folding of nascent polypeptides to refolding of mature misfolded proteins, chaperones or heat-shock proteins assist in maintaining the necessary abundance of folded proteins, compensating for the selective costs of erroneous protein synthesis, misfolding, and sequestration of proteins in NF-PPIs. In three domains of life, chaperones are essential components of protein homeostatic machinery. Chaperonins, like GroEL, effectively catalyze the folding process by increasing the rate at which misfolded proteins are converted into their folded conformations [11]–[13]; this process can lead to diminished aggregation and NF-PPIs due to the limited presence of aggregation-prone misfolded species in the cytoplasm. Lindquist and others posited that chaperones may act as phenotypic capacitors by buffering the fitness effects of deleterious mutations [14], leading to a greater genetic diversity and speeding up adaptive evolution [15], [16]. A recent in vivo study from our lab [12] also showed that the chaperone action in dynamic cellular milieu can be pleiotropic, i.e. it extends beyond the immediate effect of protein folding by reducing the participation of destabilized proteins in NF-PPIs and affecting their accessibility to ATP-dependent proteases .

Apparently, chaperones play a key role in sculpting the fitness landscape of organisms. However, understanding the evolutionary implications of this fact requires a multi-scale modeling that realistically represents the mechanism of chaperone action and reaches across the necessary length and time scales. Recently, we developed a multi-scale evolutionary model for population dynamics simulations [7], where the fitness (rate of division) of each cell is derived explicitly from its genomic sequence by using the physical principles of protein folding and interactions. The model provided insights into the co-evolution of molecular properties of proteins, their abundances in the cytoplasm, and their functional and NF-PPIs. Here we significantly extend this ab inito model to explicitly account for chaperone activity in the cytoplasm of model cells. The model elucidates not only the immediate pleiotropic effect of chaperone action on cellular fitness but also its long-term evolutionary consequences. We find that the chaperone activity provides a significant acceleration of adaptive evolution by minimizing the detrimental effect of protein misfolding and therefore opens new paths in sequence space for efficient and simultaneous optimization of multiple molecular traits, determining the fitness of model cells.

## Results

**Figure 1:**
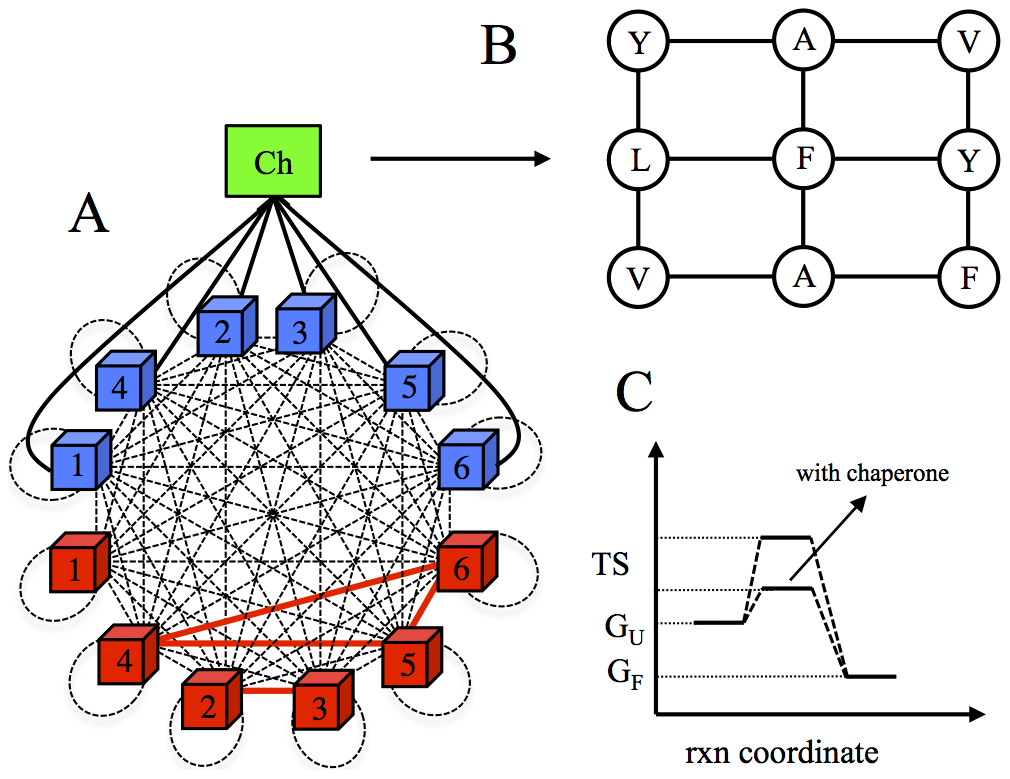
Pictorial depictions of molecular interactions, chaperone interaction surface, and free energy-reaction coordinates diagram. (A) A schematic representation of molecular interactions in the model cell. The folded (red cubes) and MG state (blue cubes) proteins in the cytosol of model cell are allowed to interact with each other to form functional (red solid lines) and non-functional (black dashed lines) interactions, which include homodimeric self-interactions (black dashed loops). Black solid lines represent the PPI network of chaperone (green square). (B) Chaperone interaction surface. A single face of cube, consisting of nine amino acid residues is used to model the interaction between chaperone and unfolded proteins. (C) Reaction (rxn) coordinate vs. free energy diagram for protein folding with and without chaperones, highlighting the catalytic activity of chaperones.

Our ab initio 6-loci model cells contain explicit genomes that encode six essential, birth rate controlling, proteins that are modeled as 27-mer lattice proteins as introduced in [7]. The advantage of this coarse-grained protein model is that a crucial conformational subset, consisting of all maximally compact conformations, can be enumerated [17], making the calculations of binding and folding stabilities exact within a selected representative conformational ensemble. At the initial stage of the simulations, each protein in the model is assigned a conformation, which is deemed folded and thus functional, and each protein complex in the functional PPI network is assumed to be functional only in one specific docking mode out of 144 possible ones [7]. The model of Ref. [7] considered NF-PPIs only between folded proteins. Here we also take into account the misfolded compact Molten Globule (MG) states of proteins [18] by modeling the ensemble of unfolded states as maximally compact yet non-native conformations (see *Methods*). As shown in Fig. 1*A*, we allow all proteins in their folded and MG states to interact with each other in the cytoplasm of model cells to form functional and non-functional protein complexes. Experimental studies show that GroEL and several other chaperones do not interact strongly with proteins in their native state, see e.g. [19]–[22]. Therefore, here we only consider interactions between the model chaperone and proteins in their MG state. As shown in Fig. 1*B*, the interaction surface of the chaperone is modeled as a 2D (3×3) lattice fragment, consisting of nine amino acid residues that are found in the apical domain of the chaperonin GroEL and that have been shown to be essential for substrate binding [23].

We assume that functional protein complexes constitute the same prototypical PPI network as in [7]: the first protein is active in monomeric form, the second and third proteins are functional as a heterodimer, and finally, the fourth, fifth and sixth proteins form a “date triangle” where they function in various combinations of pairwise complexes between them (Fig. 1*A*). We then postulate, as in [7], that the division rate of an individual cell is a product of the functional concentrations of proteins for the postulated prototypical PPI network:
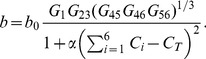
(1)Here 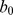 is a parameter used to scale the rate and thus the time, 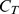 is the postulated “optimal” total concentration of proteins, which reflects the assumption that protein synthesis comes at a cost, 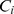 are the total concentrations of individual proteins, and 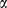 is a control parameter that defines a fitness penalty for deviation from the optimal *total* concentration of all proteins. Overall, the role of the denominator in Eq. **[1]** is to penalize the deviations from the optimal protein levels and to avoid a fitness gain by a mere overexpression of proteins. Hence, the cell division rate in our model is determined by a fitness function, which stems from an intuitive physical-biological assumption that a subset of gene products acts in concert to promote healthy cell divisions.

In what follows, we define the functional concentrations of monomer and dimers in Eq. **[1]** as
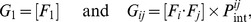
(2)where 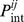 is the Boltzmann probability that proteins *i* and *j* interact with each other in a specific docking conformation (see *Methods*), 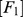 is the concentration of the monomeric protein product of gene 1 in its native folded form, 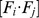 is the concentration of the binary complex formed by the folded states of proteins *i* and *j*.

We employ a simplified two-step kinetic model to describe the catalytic activity of active chaperones, as illustrated in Fig. 1*C* (see *Methods* for the technical aspects of the formulation). In this active model, the chaperone acts as a catalyst to accelerate the rate of protein folding. As a control, we also consider a passive model of chaperone action, whereby the role of chaperone is simply to bind and release proteins in their MG states. It is noteworthy that, in contrast to a conventional catalyst, which decreases the activation barrier for both forward and backward reactions, an active chaperone increases the rate of conversion of misfolded proteins into their folded form without increasing the rate of reverse reaction of unfolding. Such “one-way” catalysis, which requires consumption of ATP, increases the concentration of folded species, which is equivalent, under steady state conditions, to an effective increase of thermodynamic stability of a protein as outlined in [12]. We model binding of MG proteins to the chaperone with a pre-equilibrium assumption since the association/dissociation of chaperone with an MG protein is a fast process as compared to subsequent kinetic steps in which the actual protein folding occurs. It has been shown that these later kinetic steps, which lead to folding, are rate limiting as they almost always require ATP hydrolysis [24].

Examples of active chaperones with catalytic folding activity include the chaperonins, GroEL in prokaryotes [24] and TRiC in eukaryotes [25]. While the applicability of our model is not limited to the GroEL-like chaperonins, the catalytic activity of this class of chaperones has been well established, see e.g. [13], [24]. Therefore, our model directly applies to this class of chaperones, which forms a good experimental system to test our predictions. Henceforth, unless otherwise indicated, we refer to the chaperones with catalytic activity simply as chaperones.

### Chaperones dramatically speed up evolutionary dynamics

We explored the effect of chaperones on evolutionary dynamics by running long time evolutionary simulations (200,000 generations) of model cell populations. Our simulations start from monoclonal populations of model cells, whose sequences have been designed by using the method reported in [26] to provide high stabilities 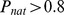 for all 6 proteins in their folded states without regard for their functional and NF-PPIs (see details in *Methods*). In our model, the acceleration of protein folding rate due to chaperone action is determined by the parameter *x*, which is the ratio of the rate at which a folded protein is released by the chaperone to the rate at which spontaneous protein folding occurs (defined in *Methods*).

To determine the effects of chaperone buffering on adaptive evolution, we tested two models – an active and a passive model. In the active model, the chaperone acts as a catalyst and accelerates protein folding. However, in the passive model, the chaperone assumes a simple role by merely binding and releasing proteins in their MG states. While for the passive model we set 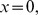 for the active model we assume a modest 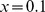 throughout this work, consistent with the estimates of the dynamic model given in [12]. In both cases we keep the chaperone concentration fixed at 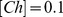. To highlight the role of chaperones we always, in parallel, run control simulations for cells without chaperones, i.e. setting 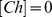.

To determine broadly the effect of chaperones on adaptation dynamics we ran evolutionary simulations at three different temperatures, i.e. *T* = 0.85 (low), T = 1.05 (medium), and T = 1.25 (high). Throughout this work, all temperatures are in units calibrated to Miyazawa-Jernigan (MJ) potentials [27].

**Figure 2:**
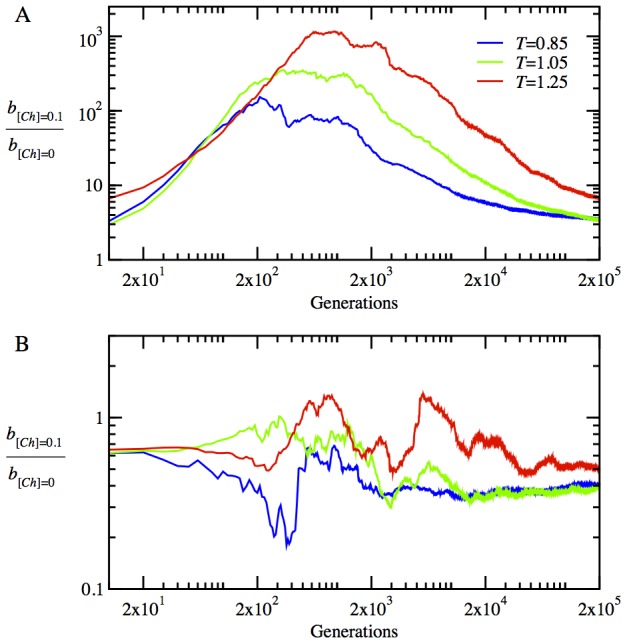
The time evolution of the fitness ratios 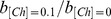 (i.e. the ratio of birth rates with chaperones and without chaperones) are presented for the active 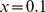 in (A) and passive 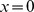 model in (B) for three different temperatures. The fitness ratios and evolutionary time are in log scale to convey the events clearly across all time scales. All data here and in the subsequent figures are ensemble averages over 100 independent stochastic trajectories.

The effect of chaperones on the evolution of fitness is presented in Fig. 2 as fitness ratio, i.e. the ratio of birth rate in the model with chaperone to that without chaperone. Fig. 2*A* shows the time evolution of 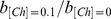 for the active model 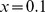. The chaperones provide dramatic fitness benefit during the adaptive evolution, especially at early stages. The effect of chaperones is more pronounced at higher temperatures, where proteins in the MG state are more prevalent. While the fitness ratio reaches its peak of 100 at intermediate adaptation times for T = 0.85, it peaks at 250 for T = 1.05 and dramatically over 1000 for T = 1.25. After the initial fast adaptation period, the relative fitness effect of chaperones abates. Up to this point, however, the cells already have gained a considerable fitness advantage, and in the long time limit, we see gradually declining fitness ratios as the organisms become more and more fit. Nevertheless, the evolutionary dynamics with chaperones always leads to a higher long-time fitness than the evolutionary dynamics without chaperones, although the final fitness ratio is not as dramatic as those observed at intermediate evolutionary times.

Fig. 2*B* shows the time evolution of 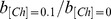 for the passive chaperone model 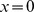. It is clear that the chaperones in the passive model do not provide a noticeable fitness gain but rather a small fitness loss (due to the sequestration of proteins by chaperones) at the initial stage of adaptation for all three temperatures. In light of these results, we conclude that the active folding of proteins by chaperones is necessary to provide a fitness benefit to cells in the evolutionary dynamics. In the following, unless otherwise indicated, we present the data only for the active model at the low temperature T = 0.85 as representative of our general results.

### Chaperones promote epistasis in the evolution of molecular properties of proteins

**Figure 3:**
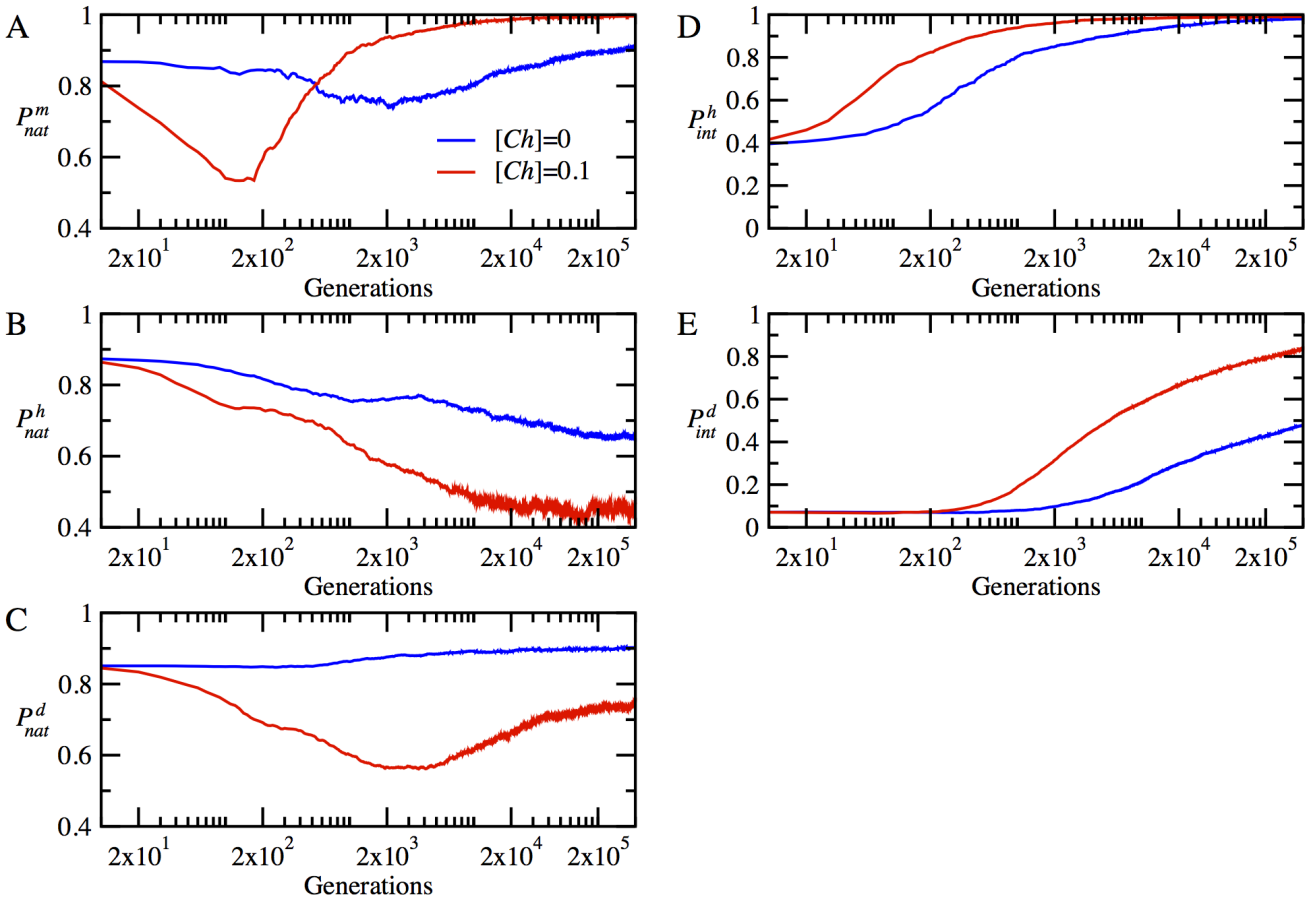
The time evolution of mean protein stabilities and mean interaction probabilities of functional dimers in the absence and presence of chaperones, i.e. for 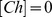 (blue lines) and 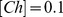 (red lines), respectively, at temperature *T* = 0.85. (A) The time evolution of stability 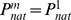 for monomeric proteins. (B) The time evolution of mean stability, 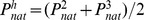 for heterodimer proteins. (C) The time evolution of mean stability, 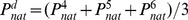 for date triangle proteins. (D) The time evolution of interaction probability, 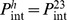 for the heterodimer complexes. (E) The time evolution of mean interaction probability, 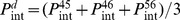 for the date triangle complexes.

Now, we turn to a detailed account of the evolutionary dynamics of the physicochemical properties of proteins, i.e. their stabilities 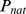 and functional interaction probabilities 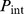 for the functional heterodimers and date triangles (see *Methods* for the definitions of these quantities). We present the time evolution of 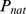 for the monomer in Fig. 3*A*, for the heterodimer proteins in Fig. 3*B*, and for the date triangle proteins in Fig. 3*C*. The chaperones provide a noticeable increase in stability for the monomeric proteins, as seen in Fig. 3*A*. Interestingly, at the initial stage of adaptation within 500 generations, the monomer loses its stability considerably by accumulating destabilizing mutations in the presence of chaperones. However, subsequent mutations bring about a rapid turnaround, resulting in a very stable monomer, which persists throughout the rest of the evolutionary dynamics. The non-monotonic dependence of stability of the monomeric protein on evolutionary time is an indication of a chaperone-enhanced epistatic behavior. The chaperone buffering relaxes significantly the stability constraint and allows the accumulation of more mutations in the locus encoding natively monomeric protein. This effect is mainly responsible for the initial sharp drop in the stability of the monomer. The resulting enhanced genetic diversity provides a path to a faster optimization of collective properties of all proteins in the cytoplasm such as NF-PPIs, as we show below.

The evolutionary dynamics of stability for the heterodimer and date triangle proteins show quite a different trend, as seen in Figs 3*B* and 3*C*. Initially, both the heterodimer and date triangle proteins lose their stability, but later on, the stability of date triangle proteins is slowly restored. However, in striking contrast to the monomer, the stability of heterodimer and date triangle proteins in the presence of chaperones shows a downward trend with evolutionary time, as compared to that of the chaperone-free cytoplasm of model cells.

The evolution of strengths of functional interactions, reflected in the parameter 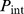 for the heterodimer and date triangle proteins, is given in Fig. 3*D* and 3*E*, respectively. The chaperones significantly increase 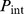 for both the heterodimer and date triangle complexes. 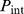 for the heterodimer increases rapidly within first 1000 generations, in the presence of chaperones. The rate of increase of 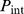 for the date triangle is slower than that for the heterodimer; nevertheless, the chaperones provide a significant increase in 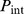 for the date triangle complexes as well. Hence, our results show that the chaperones shift the balance between the strengths of functional interaction and stability of proteins in favor of the former at the expense of the latter. Indeed, it is more advantageous for faster adaptation that the heterodimer and date triangle proteins primarily develop strong interaction surfaces to contribute to the fitness. High stability of proteins establishes later on once the strong functional interaction between them is ensured.

### Chaperones dramatically diminish the loss of proteins to NF-PPIs

**Figure 4:**
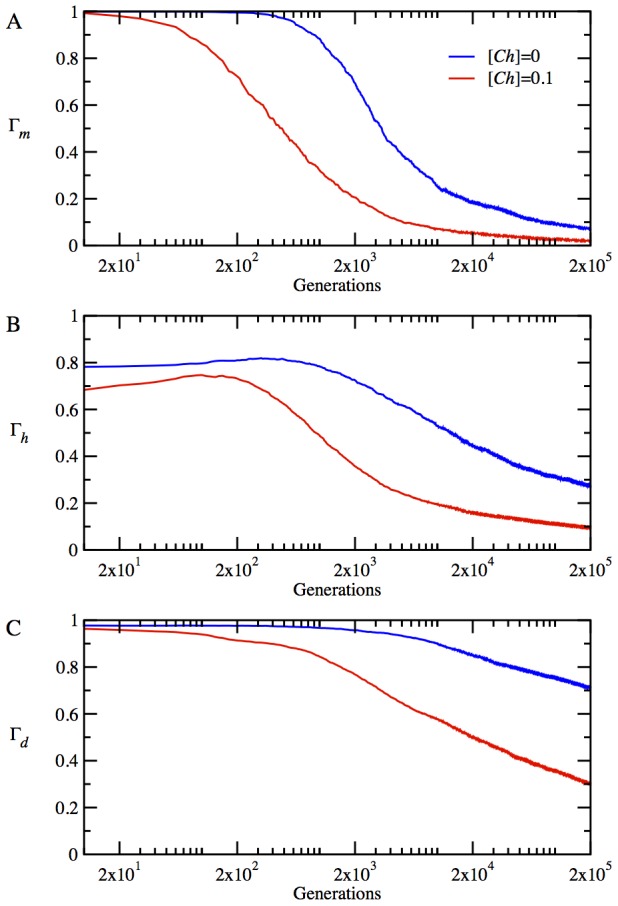
The time evolution of the mean value of the fractions of proteins involved in NFP-PPIs to their total concentrations for 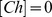 (blue lines) and 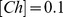 (red lines), at temperature *T* = 0.85. (A) NF-PPI for functional monomer 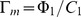 (B) average NF-PPI for heterodimers 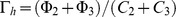 and (C) NF-PPI for date triangles 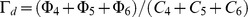 where 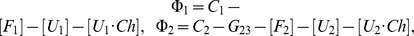 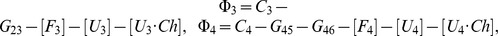 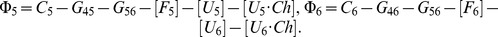

While the effect of chaperones on protein stabilities and interactions is significant, it cannot fully account for the huge overall fitness increase, which transiently reaches up to a factor of 100 at the low temperature T = 0.85 (see Fig. 2*A*). Therefore, there must be another factor affecting fitness, where the effect of chaperones appears even more pronounced. To that end, we turn to the analysis of NF-PPIs, which affect fitness through modulation of concentrations of proteins in their functional form. We find that at the early stages of adaptation the chaperones dramatically decrease the concentrations of protein complexes engaged in NF-PPIs, releasing more proteins to become functional. This can be seen in Fig. 4, where we plot the time evolution of the fraction of protein material wasted in NF-PPIs in the absence and presence of chaperones. Specifically, we present the time evolution of 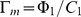 for the monomeric protein and 
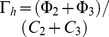
 for the heterodimers and 
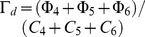
 for the date triangles, where 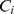 is the total concentration protein *i* and 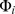 is the total concentration of protein *i* involved in NF-PPIs. Fig. 4 shows that the vast majority of proteins are lost to NF-PPIs at the beginning of evolutionary runs, where the sequences are optimized for stability only without regard for functional PPIs. Apparently, at the very early stage of adaptation, cell resources are mostly wasted unproductively to NF-PPIs. Both Fig. 4*A* and 4*B* show that the chaperones give rise to a rapid increase in the functional concentrations of monomeric and heterodimer proteins within the first 5,000 generations. As shown in Fig. 4*C*, the rate of decrease of NF-PPIs for the date triangle proteins is slower than that observed for the monomer and heterodimer proteins; nevertheless, with chaperones, it still occurs at the early stage of adaptation within 10,000 generations.

We find therefore that, while the chaperones interact directly with proteins to affect its molecular properties, their greatest impact on cellular fitness occurs indirectly through the optimization of a collective property of all proteins in the cytoplasm of model cells, namely, their NF-PPIs.

### Chaperones speed up evolution by promoting neutrality and polymorphism

Our results indicate that the chaperones significantly accelerate the rate of adaptive evolution. Customarily, a well-known parameter 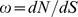, where 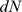 and 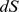 are the non-synonymous and synonymous substitution rates, respectively, represents a quantitative measure of evolutionary rate. A straightforward approach to calculate 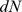 and 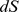 at any time step in simulation is to compare the genome of the dominant clone in the population to the initial starting genome. However, we find that this approach is problematic for our model in the long time limit when multiple substitutions at a single site become frequent. Here, we employ a slightly different approach. Following Wilke [28], we define the evolutionary rate as 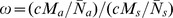 where 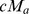 and 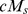 are the cumulative non-synonymous and synonymous substitution counts summed over short time intervals of 100 generations, and 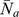 and 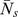 are arithmetic means of weights for non-synonymous and synonymous sites, which account for different degeneracies of codons in the genetic code, calculated over time frames of 100 generations, see *Methods* for details.

**Figure 5:**
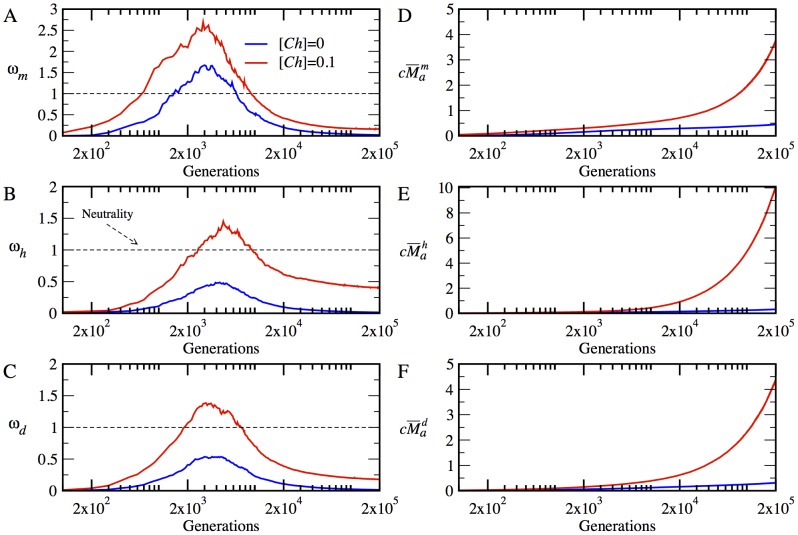
The time evolution of mean 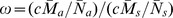 and 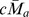 in the absence and presence of chaperones, i.e. for 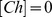 (blue lines) and 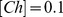 (red lines), respectively. The dashed line at 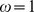 represents the neutral evolution. The time evolution of 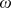 is plotted in (A) for the monomer 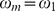, in (B) for the heterodimer 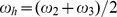, and in (C), for the date triangle 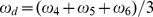. The time evolution of 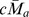 is plotted in (D) for the monomer 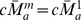, in (E) for the heterodimer 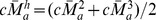, and in (F) for the date triangle 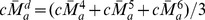.

We summarize our results, averaged over multiple evolutionary runs, in Fig. 5*A* to 5*C* for 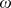 to highlight the type and magnitude of selection acting on different proteins at different stages of adaptation. In Figs., from 5*D* to 5*F*, we present the cumulative weighted non-synonymous substitutions 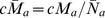 for different types of proteins in our system. Further, in Fig. *S1*, we provide the synonymous substitution rates 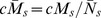. The evolutionary dynamics of 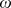, 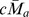 and 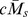 for individual trajectories are also given in Fig. *S2*.

We found that at the very early stage of adaptation, after 500 generation, the chaperones induce a strong positive selection pressure on the monomer, which lasts, in average for about 10000 generations, after which the monomer falls under purifying selection. However, without the chaperones, the monomer evolves under positive selection only for a short time between 1500 to 6000 generations. On the other hand, without the chaperones, the net selection on both the heterodimer and date triangle genes is negative, apparently due to the dominance of the stability constraint. In the presence of chaperone buffering, however, these loci evolve under positive selection for about 8000 to 10000 generations before they revert back to purifying selection. An important generic effect apparent in the time evolution of 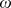 is that the chaperone buffering relaxes the negative selection pressure on all proteins and promotes the fixation of a greater number of beneficial mutations. Therefore, after the initial stage of fast adaptation, when all genes evolve under positive selection, we observe that the chaperones bring all genes closer to neutral regime in the adapted populations.

**Figure 6:**
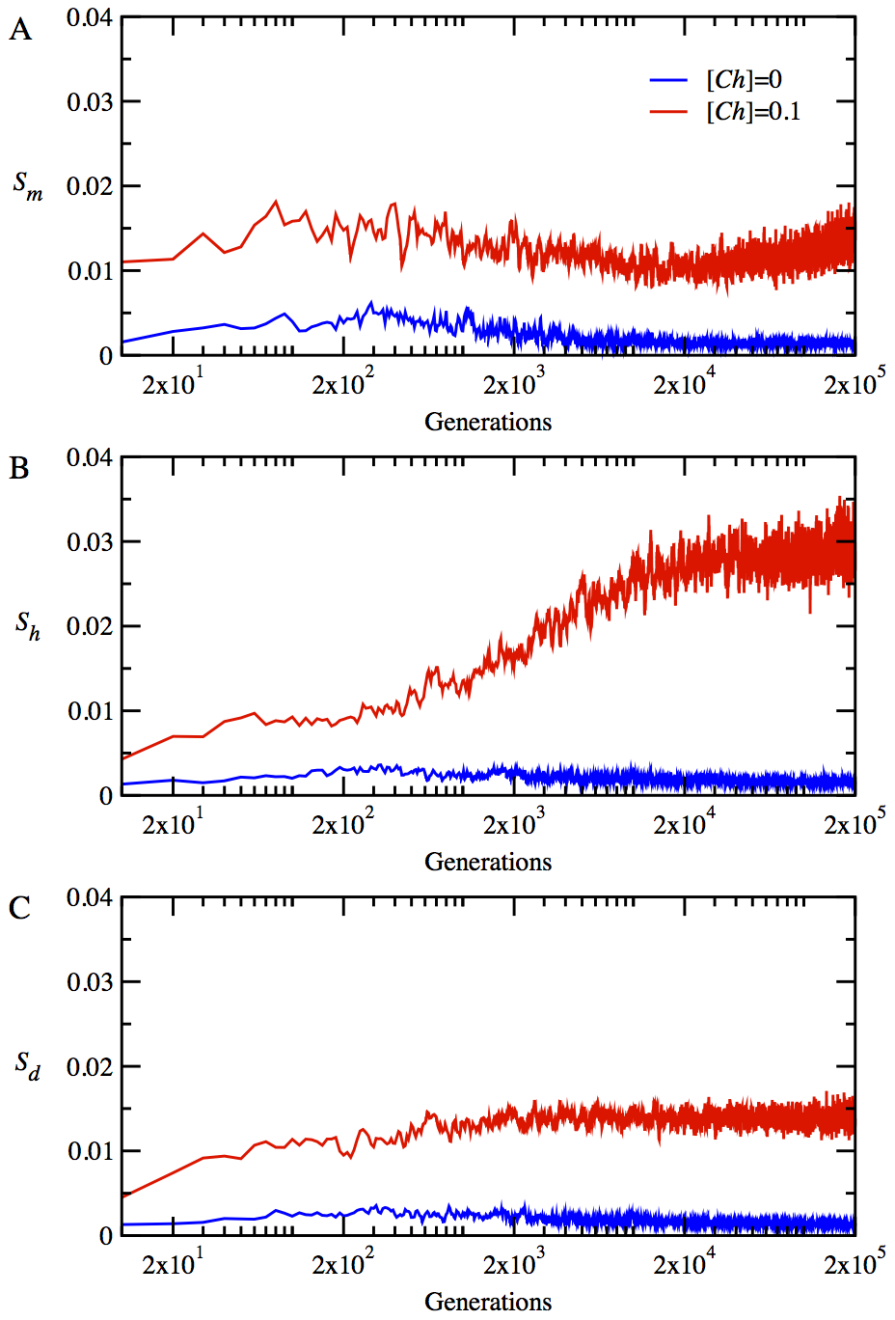
The time evolution of mean sequence entropy is plotted in the absence and presence of chaperones, i.e. for 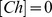 (blue lines) and 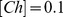 (red lines), respectively, at temperature *T* = 0.85. The time evolution of mean sequence entropy is given in (A) for the monomer 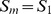, in (B) for the heterodimer 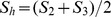, and in (C) for date triangle proteins 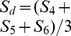.

Next, we evaluated the effect of chaperones on the polymorphism in evolving populations of model cells. To that end we determined the average sequence entropy for each protein locus in our model. This quantity is determined from the alignment of gene sequences between all model organisms within the population (see *Methods*). These results are presented in Fig. 6.

Overall, we find that chaperones greatly enhance polymorphism in evolving populations. For all protein types, the sequence entropy rapidly increases within a few hundred generations with chaperone. For the monomer and date triangle proteins, the entropy stays approximately at the same level for the duration of an evolutionary run after the initial fast increase. For the heterodimer proteins, however, the entropy gradually increases reaching a level, which is almost two times higher than that for the monomer and date triangle proteins. A greater degree of polymorphism observed for the heterodimer proteins helps these proteins evolve faster than other loci in the model, as we note below.

The enhanced neutrality due to chaperone buffering also increases the rate of protein evolution considerably. Indeed, as seen in Figs., from 5*D* to 5*F*, the chaperones increase the net number of non-synonymous mutations for all loci. Initially, the monomer still evolves with the chaperones faster than the heterodimer and date triangle genes. However, the rate of evolution of heterodimer is the fastest as a result of more phenotypic diversity of this gene in population as indicated by the entropy plot (see Fig. 6*B*). Apparently, the evolutionary rates of the heterodimer and date triangle loci are slower that that of the monomer throughout the evolutionary dynamics with or without chaperones. Finally, Fig. *S1* shows that the rate of synonymous substitutions is approximately the same for all protein types, as could be expected. However, we also see that the rate of synonymous substitutions is slightly faster with chaperones as compared to that of chaperone-free evolution. Such slightly faster evolution of synonymous substitutions might be due to hitchhiking of neutral mutations with beneficial ones that should more pronounced with chaperone evolution.

## Discussion

Our ab intio cell model, while much simpler than real biological systems, captures the essence of biological complexity that stems from the fact that the main effects, epistatic effects, and pleiotropic effects on different parameters often act in antagonistic directions. The pleiotropic concept of optimization of antagonistic traits in evolutionary biology, which gives rise to a complex fitness landscape, has its analog in the concept of frustration in physics, where competing interactions lead to a complex energy landscape with many suboptimal minima equal to or close to global minimum [29], [30]. In our model, the molecular traits, whose optimization might be antagonistic, include protein stability, abundances, functional, and NF-PPIs. An earlier study showed how antagonistic constraints result in a peculiar co-evolution of protein abundances and functional PPIs [7]. Here we introduced a new essential component of the cellular milieu – the chaperone activity, which enhances the conversion of proteins from the MG state to their native conformation. The chaperone action in our model partially relaxes an essential constraint on protein sequences to maintain high stability of proteins. The resulting chaperone buffering dramatically affects evolutionary dynamics by opening up sequence space, to provide a dramatic acceleration of the adaption process. We find that only the active chaperone model has a strong effect on evolutionary dynamics, while the passive chaperone model, where an MG protein is bound to the chaperone to prevent its sequestration to NF-PPIs has no effect on fitness (Fig. 2*B*). However, an important caveat here is that our model does not consider an irreversible aggregation and other elements of protein quality control such as proteolytic activity. Hence, the passive model might also be efficient when all the kinetic aspects of protein quality control are taken into account.

Mechanistically, our main finding is that the chaperones act pleiotropically to affect fitness in a number of ways. Firstly, the relaxation of the stability constraint allows achieving stronger functional PPIs at the expense of lower thermodynamic stability for the proteins participating in the functional PPIs (Fig. 3). Secondly, a more dramatic effect of the chaperone on cellular fitness stems from faster and greater decrease of the NF-PPIs in the course of the evolutionary dynamics (Fig. 4). The NF-PPIs are a collective property of all proteins in the cellular milieu. There is evidence that proteins in their MG state are largely responsible for NF-PPIs [31]. The active chaperone in our model converts the proteins in their MG states into their folded conformations, leading to a drop in NF-PPIs with an ensuing increase in fitness due to a diminished sequestration of functional proteins.

Recent experimental and theoretical studies with the chaperonin GroEL corroborate some of our findings. Tokuriki and Tawfik performed a series of random mutagenesis experiments on a number of non-endogenous enzymes expressed in *E. Coli* to investigate the impact of overexpression of GroEL on enzyme evolution [16]. They found that GroEL indeed helps folding of destabilized proteins and potentially facilitates the evolution of enzymes to gain new functions. The acceleration of adaptive evolution by GroEL is also found in a recent study [15] in which the evolutionary rates of GroEL clients and non-clients [32] were compared. It was found that the GroEL obligatory proteins evolve 35% faster than the proteins that fold spontaneously without the GroEL assistance [15]. The importance of GroEL for adaptive evolution is highlighted by the case of the endosymbiotic bacterium *Buchnera*, which often undergoes population bottlenecks through maternal transmission and thus quickly accumulates random mutations that destabilize proteins [33]. Remarkably, the expression level of GroEL in *Buchnera* is almost 8 times greater than that of *E. coli* under the normal conditions.

Quayle and Bullock define evolvability as the number of generations that it takes for a population to reach its phenotypic target that maximizes fitness [34]. Our study highlights the dual role of chaperones not only as a catalyst of protein folding but also as a catalyst on the fitness landscape, which lowers the genetic “barriers” between phenotypes and thus promotes evolvability. A key prediction emerging from our analysis is that the catalytic activity of chaperone gives rise to a dramatic acceleration of adaptive evolution. Hence, we predict that the depletion of active chaperones through down-regulation of their expression should directly affect the rate at which organisms adapt to new environments, which can be directly experimentally testable. This work is currently in progress in our lab.

## Methods

### Protein stability and interactions

Our proteins consist of 27 amino acid residues that fold into 3×3×3 cubic lattice conformations [17]. We use the MJ potentials to model intra- and inter-molecular interactions [27]. While the 27-mer lattice model has 103,346 maximally compact conformations [17], we employ a uniform subset of randomly selected 10,000 conformations as our conformational ensemble to speed up calculations [7]. We calculate the Boltzmann probability of folding to a native state, i.e. 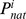 for each protein 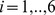 as follows
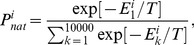
(3)where 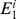 is the energy of the native conformation and 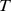 is the temperature in units corresponding to the calibration with MJ potentials.

We model the functional protein-protein and protein-chaperone interactions using a rigid docking scheme. The six faces of a cubic lattice provide six possible interaction surfaces and there are four rotational degrees of freedom to dock two interaction surfaces of two lattice proteins. Hence, in total, there exist 6×6×4 = 144 docking modes for a binary protein complex. The Boltzmann probability of interaction 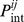 between the dimer proteins *i* and *j* are calculated as
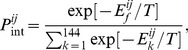
(4)where 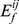 is the interaction energy of the functional binding mode, 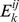 are the interaction energies for 144 docking modes and 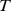 is the temperature. Because 27-mers are quite small (as compared to real protein sizes), in order to represent both interior and interaction surface of proteins, we employed two different lattice conformations to model our proteins: one for interior part that determines stability and one for interaction surface that determines PPI, as has been done in previous studies, see e.g. Ref. [7]. Hence, the lattice conformations that we used to represent protein surfaces in order to calculate 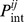 are randomly chosen conformations but they are not the same lattice conformation that we used to represent the stability energetics of native folds. This approach provides a less tight coupling between interior and exterior of proteins that would be the case for small 27-mers representing therefore a more realistic description of protein geometry and energetics.

### The model of active chaperone action

In the absence of chaperones, the folded state and the unfolded ensemble of states (which also includes compact MG states) for any protein “*i*” are at equilibrium, satisfying detailed balance with the corresponding folding 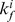 and unfolding rates 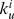:
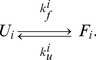
(5)


The active chaperone changes this picture dramatically. In general, the operation of chaperones requires input energy by ATP hydrolysis. The energy flux due to the ATP hydrolysis by chaperone causes the violation of detailed balance between the folded and unfolded forms of a protein. Therefore, following the findings in [19]–[22], we assume that the chaperon 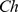 interacts with a protein in its misfolded MG conformation 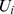 to form a pre-equilibrium dimer complex 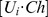, from which the protein is released in its folded form 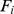,
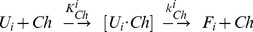
(6)where 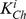 are the pre-equilibrium constants for the chaperone-unfolded protein complex, and 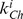 are the rate constants for the chaperone assisted folding. While the native state is uniquely defined by a single conformation in our model, the unfolded states constitute an ensemble of conformations, which we take into account as a representative ensemble via a mean field approximation (see below).

The steady state solution of Eqs. 5 and 6 leads to the following,
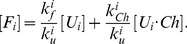
(7)By introducing the ratio of the rate constant for chaperone assisted folding to the rate constant for unassisted folding, i.e. 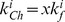 and also by making use of the following two equilibrium relations,
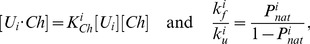
(8)we arrive at the equation,
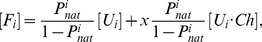
(9)where 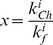 is the ratio of the rate of release of native proteins from the chaperone complex to the rate of spontaneous folding.

### Stochastic simulation algorithm

Our simulations start from initial sequences designed to be stable in their respective native conformations 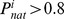; the PPIs of initial sequences were not optimized. We randomly assigned the functional docking modes for the heterodimers and date triangles. In order to keep protein folds fixed throughout the simulations, we discarded the cells encoding proteins whose assigned native folds were no longer the lowest energy configurations. A constant population size of *M* = 1000 is maintained throughout the simulations.

We use a variant of the Gillespie algorithm to generate stochastic evolutionary trajectories in our simulations. Using two uniformly distributed random numbers, 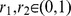, the algorithm decides when and which cell undergoes a cell division. Given the normalized birth rates as 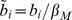 where 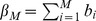 for each cell *i* in a population of size 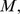 we define the cumulative probabilities as 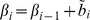. Note that 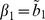 and 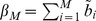. The waiting time *dt* for the next cell division to occur at time *t+dt* is determined by 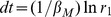. The cell “*i*” divides when the second random number falls in the interval 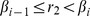.

Upon cell division, a mother cell gives birth to a daughter cell. A newborn cell replaces a randomly chosen cell in the population in order to maintain constant population size. Also, whenever a mutation changes the lowest energy protein fold or hits a stop codon, such cells are discarded from the population. Upon semi-conservative replication, both the mother and daughter cells are subject to either a mutational event with constant probability *m* = 0.001 per gene per replication (whereby a nucleotide is randomly changed) or the expression level of one randomly chosen protein in a cell can change with a constant rate *er* = 0.01 per cell division such that the concentration of a protein *i* in a daughter cell is derived from that of a mother cell as follows 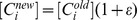 where 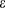 is a Gaussian random number with zero mean and variance 0.1. At the beginning of our simulations, we set the concentrations of each protein and chaperone equally at 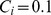.

### A mean field approximation for equilibrium constants

Six proteins encoded in our cell model make four *functional* interactions in total. In addition, we allow all possible *non-functional* interactions between all proteins in their folded as well as MG conformations (see Fig. 1*A*). More specifically, we consider the equilibrium reactions, forming homo- as well as heterodimers between the folded proteins,
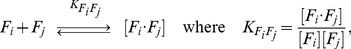
(10)between the folded and unfolded proteins,
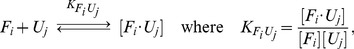
(11)and between the unfolded proteins,
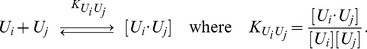
(12)Next, we discuss how we calculate the equilibrium constants for protein-protein and protein-chaperone interactions. We use the index set 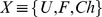 for different molecular species, the subscripts *i* and *j* for different proteins, and the superscripts *n* and *m* for different protein conformations. Given the two conformations *n* and *m* of lattice proteins *i* and *j*, the binding constant can be written as
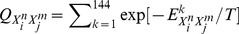
(13)where 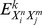 are the interactions energies and 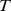 is the temperature.

In order to reduce computational cost in calculating the binding equilibrium constants, we make use of a mean field approximation in which we choose 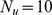 representative MG conformations randomly out of 10,000 conformations and assume that each of these conformations is equally likely to occur in the MG ensemble. In what follows, we calculate the binding equilibrium constants for the dimers formed by the folded proteins exactly as
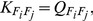
(14)and the dimers formed by the folded and unfolded proteins as
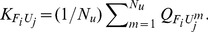
(15)The binding equilibrium constants for the heterodimers, i.e. 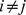, formed by the unfolded proteins are calculated by
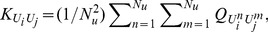
(16)and the homodimers formed by the unfolded proteins are given by
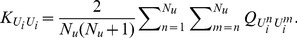
(17)To model the protein-chaperone interactions, we use a 3×3 square lattice face to mimic an interaction surface for the chaperone (See Fig. 1*B*). For the protein-chaperone interactions, there are 1×6×4 = 24 docking modes. Hence, the binding constant for an unfolded protein *U_i_* with conformations *n* and the chaperone 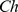 is of the form,
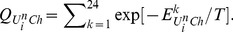
(18)By using our mean field approximation, the pre-equilibrium constant for the association of chaperone with an unfolded protein is given by
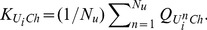
(19)The conservation of mass for each protein 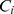 and chaperone 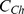 in our system can be written as
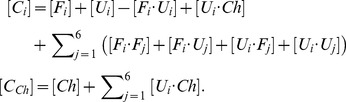
(20)


### An iterative method to solve the LMA equations

Due to the non-linear nature of the law of mass action (LMA) equations, a direct integration of these coupled equations may only be possible for small systems. However, by using iterative algorithms, the LMA equations can easily be solved even for large systems. Previous iterative algorithms were developed to solve the LMA equations involving only equilibrium reactions between different molecular species. The LMA equations in our system involve not only different molecular species but also equilibrium reactions between conformational isomers of the same molecular species and therefore may not be solved by the existing algorithms, see e.g. [35]. Here, we present a straightforward generalization of the existing iterative algorithms.

We start our iterative algorithm by initializing the concentrations of monomeric unfolded proteins and chaperone, i.e. 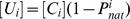 and 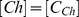. Our algorithm consists of iterations of three sets of equations to find equilibrium concentrations of all chemical species in our system. First, by substituting 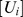 and 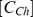 into the right hand side of Eq. [**9**] we determine 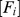. Second, by using the new value of 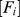 along with 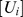 and 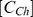 we calculate the two quantities:
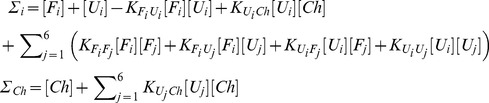
(21)Third, we find the new values of 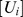 and 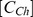 by using,
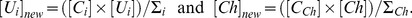
(22)By updating the old values of 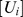 and 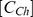 with the new values, i.e. 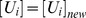 and 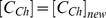, and substituting them back into Eq. **[9]**, we continue our iterations until the error threshold 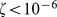 is achieved where 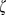 is defined by
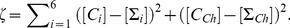
(23)


### Calculations of sequence entropy

In order to determine the degree of polymorphism in a population, we calculated the sequence entropy for each protein 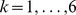 by using the formula
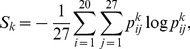
(24)where 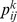 is the frequency of amino acid type “*i*” in the *j*th position in the multiple alignment (among all cells in the population) of sequences of a protein “*k*”.

### Calculations of 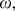 synonymous and non-synonymous substitution rates

In calculation of 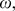 we used the following formula 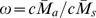, where 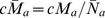 and 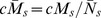 are the normalized cumulative non-synonymous and synonymous substitutions rates, and 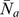 and 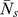 are the arithmetic mean of non-synonymous and synonymous substitutions reflecting the instant composition and degeneracies in the genetic code [28]. In order to calculate the quantities 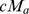, 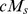, 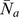 and 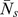 we partitioned the overall simulation time into the time intervals of length 100 generations. Given the two DNA sequences, say, DNA-1 and DNA-2 that are 100 generations apart, we first count the number of synonymous 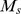 and non-synonymous 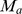 substitutions between them, and second determine the number of synonymous 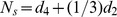 and non-synonymous 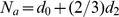 sites at the frames in this time interval, where 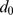 is non-degenerate, 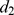 is 2-fold and 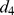 4-fold degenerate sites, respectively [36]. By using the above quantities, we next calculate the cumulative sums 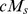 and 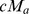, over all time intervals and the arithmetic averages 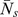 and 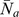, and finally determine 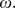


## Supporting Information

Figure S1The time evolution of 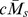 is given in the absence and presence of chaperones, i.e. for 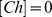 (blue lines) and 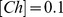 (red lines), respectively. The time evolution of 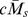 is plotted in (A) for the monomer 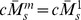, in (B) for the heterodimer 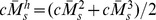, and in (C) for the date triangle 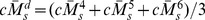. All results are ensemble averages over 100 independent stochastic trajectories.(TIFF)

Figure S2The time evolution of 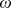, 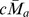 and 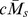 for the locus encoding functional monomeric protein (#1) is plotted for 5 different individual stochastic trajectories. Each color marks an individual trajectory. The time evolution of 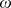, 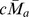 and 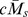 is plotted in subfigures (A), (B), and (C), respectively, for chaperone-free evolution. The time evolution of 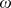, 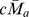 and 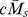 is plotted in subfigures (D), (E), and (F), respectively, for evolution with chaperones.(TIFF)
