# Supplementary figures and images for: Catalysis of Protein Folding by Chaperones Accelerates Evolutionary Dynamics in Adapting Cell Populations
Source: PLoS Comput Biol. 2013 Nov 7;9(11):e1003269. doi: 10.1371/journal.pcbi.1003269 (PMC3820506; doi:10.1371/journal.pcbi.1003269)

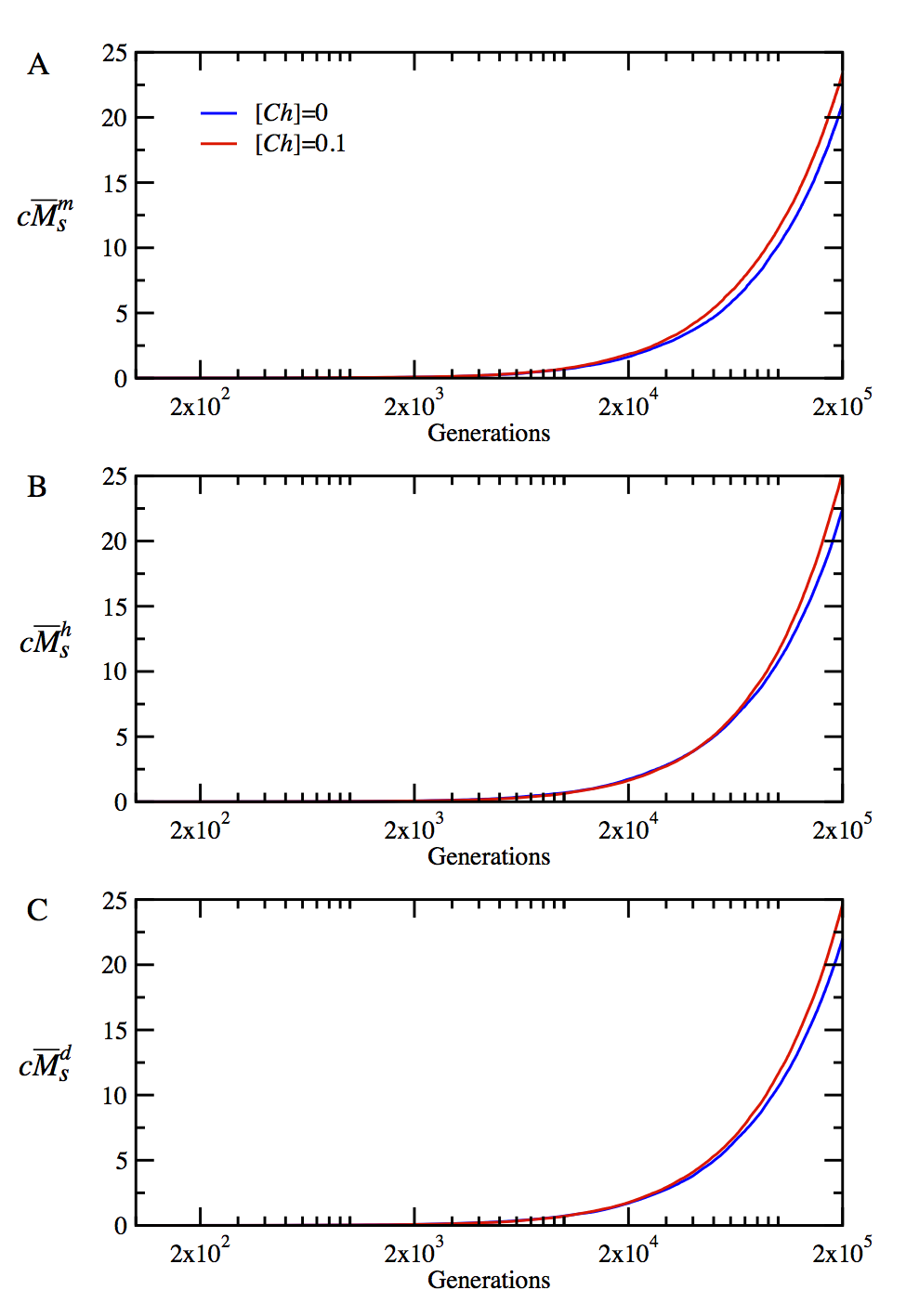

Supplement: Figure S1 — The time evolution of is given in the absence and presence of chaperones, i.e. for (blue lines) and (red lines), respectively. The time evolution of is plotted in (A) for the monomer , in (B) for the heterodimer , and in (C) for the date triangle . All results are ensemble averages over 100 independent stochastic trajectories. (TIFF) [file pcbi.1003269.s001.tiff]

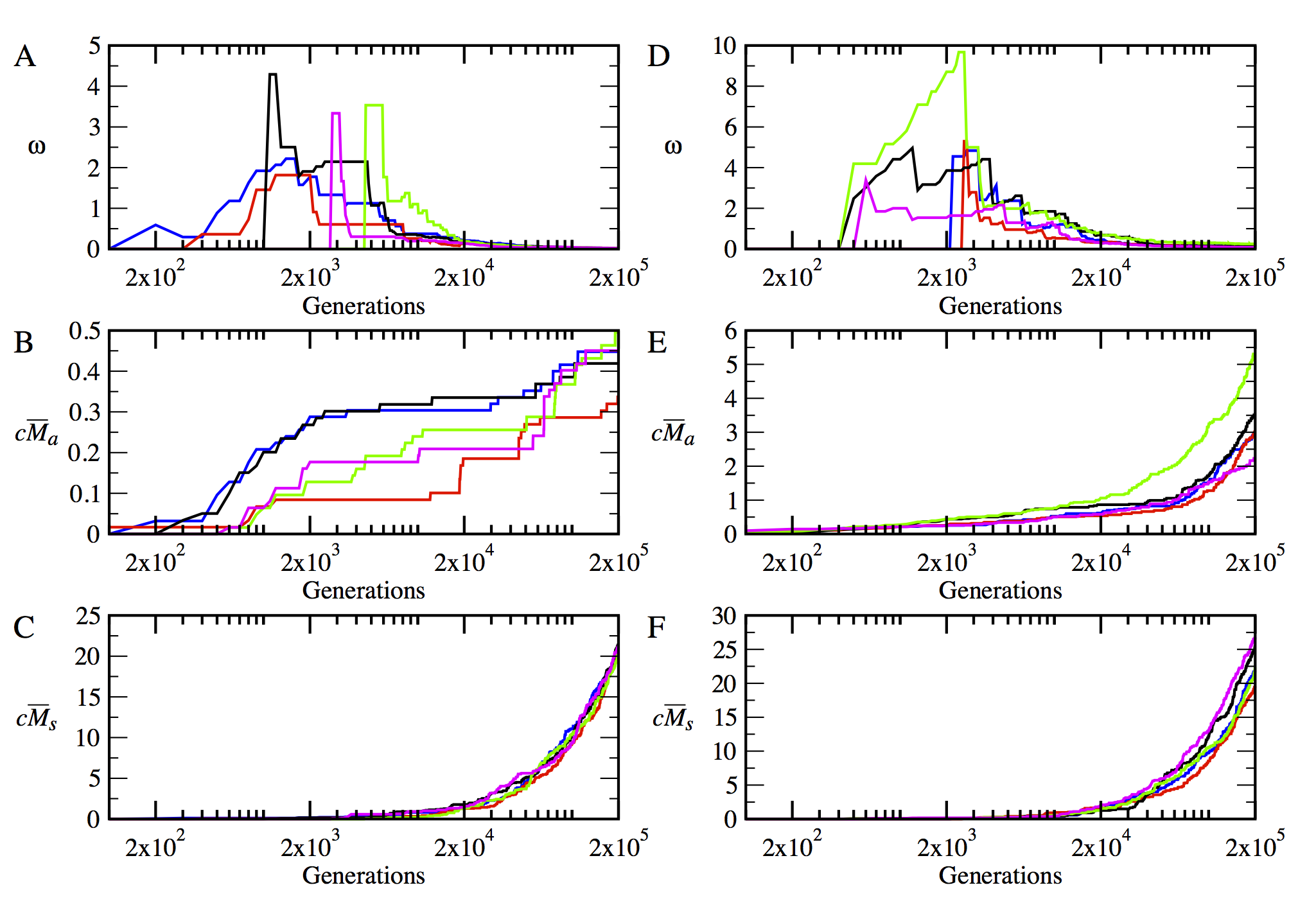

Supplement: Figure S2 — The time evolution of , and for the locus encoding functional monomeric protein (#1) is plotted for 5 different individual stochastic trajectories. Each color marks an individual trajectory. The time evolution of , and is plotted in subfigures (A), (B), and (C), respectively, for chaperone-free evolution. The time evolution of , and is plotted in subfigures (D), (E), and (F), respectively, for evolution with chaperones. (TIFF) [file pcbi.1003269.s002.tiff]
